# Designing prepacked aggregate concrete for improved mechanical properties and its field application in constructing steel tube concrete

**DOI:** 10.1038/s41598-024-65295-8

**Published:** 2024-06-25

**Authors:** Xiaojun Zhou, Shiming Bai, Yingda Zhang, Lin Xie, Xianliang Zhou

**Affiliations:** School of Architecture and Civil Engineering, Xihua University, Chengdu, 610039 China

**Keywords:** Prepacked aggregate concrete, Compactness, Grouting method, Ultrasonic pulse velocity, Steel tube concrete, Engineering, Civil engineering

## Abstract

Prepacked aggregate concrete (PAC) is made by placing coarse aggregates of various sizes into a formwork and then filling the voids between coarse aggregate and grout. The mechanical performance of PAC is dominated by the compactness due to grout filling, but few study considered the pouring methods and grout performance synchronously. The coupled effect of pouring methods and grout performance on the compactness of PAC is investigated in this study. The results show that the gravity pouring method is only suitable for grouts with good flowability. The pump pouring method is more widely used. It can be adapted to grout with poorer fluidity and coarse aggregate with greater apparent density. The ultrasonic pulse velocity test method provides a relatively accurate evaluation of the compactness of PAC. Furthermore, due to the enhanced mechanical properties of PAC, the filed application potential in the preparation of steel tube concrete columns has also been confirmed, where the results exhibited that PAC based steel tube concrete contributed to an enhanced ductility and autogenous shrinkage.

## Introduction

Prepacked aggregate concrete (PAC) is a special type of concrete formed by placing and packing appropriately graded coarse aggregates in a formwork and then filling the voids between the coarse aggregates with grout^1–3^. Due to its unique forming method, PAC is also known as "two-stage concrete"^4,5^. PAC is convenient for construction and in line with the low-carbon, green development outlook. It is particularly suitable for complicated and densely reinforced concrete structures or situations where conventional concrete pouring is challenging^6^. PAC has been widely applied in underwater concrete, large-volume concrete construction, and reinforcement in bridge and tunnel engineering^7–10^. The reduction in cement dosage in PAC has alleviated the pressure of excessive resource extraction and utilization, demonstrating significant economic and environmental benefits^10–12^. PAC also exhibits excellent mechanical properties, including reduced concrete shrinkage, increased concrete stiffness, and improved impermeability^13,14^.

Currently, there is limited research on the relationship between the flowability, pouring methods, and pouring density of different types of grouting materials. Unlike traditional concrete, PAC employs high-flow grouting materials, avoiding the need to mix coarse aggregates with a certain thickness of mortar to enhance fluidity, as well as the traditional method of compacting concrete through vibration^15^. The construction methods for PAC primarily include gravity pouring and pump injection^1,4,16–18^. In gravity pouring, the casting material is directly poured onto the surface of pre-filled aggregates, relying on the weight of the casting material to fill the voids among coarse aggregates^19^. While gravity pouring is a common method for PAC construction, it presents potential issues such as inadequate pouring uniformity. This is due to its reliance on the self-weight of the casting material to fill the gaps among coarse aggregates, potentially leading to uneven compaction within the structure and impacting the overall performance of the concrete. The particle size of the coarse aggregates also significantly affects the density of the grouting material. If the particle size is too small, the grouting material fills slowly and has poor filling density or even local voids^20^. Najjar^21^ noted that the selection of grouting methods is directly related to the minimum particle size of the coarse aggregates. Gravity grouting has been successfully applied to aggregates with a minimum particle size of 50 mm. On the other hand, pump injection utilizes pump pressure to push grouting material upward from the bottom to fill the voids among coarse aggregates^4^. Compared to gravity pouring, pump injection is more widely applied in practical engineering and is subject to specific technical requirements in regulations. However, the broader application of pump injection in prepacked aggregate concrete is constrained by higher demands on mechanical equipment and operational techniques, limiting its widespread adoption and utilization. Kunal K. Das^22^ studied gravity pouring and proposed that for coarse aggregates, a size of 20 mm or above is usually needed for the gravity method to achieve a good grouting effect. If smaller coarse aggregates are used, pump injection should be considered to fill small pores. However, in PAC large-volume concrete construction, the minimum particle size of the coarse aggregates is determined by the fluidity of the grouting material. The better the fluidity of the grouting material is, the smaller the minimum particle size that can be used. The fluidity of the grouting material and the particle size and shape of the coarse aggregates are important factors affecting the performance of prepacked aggregate concrete^23,24^. When the fluidity of the grouting material is controlled at 18 ± 2 s, the density of PAC formed by gravity pouring and pump injection is similar to that of conventional concrete. When the fluidity of the grouting material decreases, the density of the specimens formed by gravity pouring also decreases.

Besides, PAC has low shrinkage but higher brittleness compared to conventional concrete, while steel pipes have good ductility^25,26^. Combining prepacked aggregate concrete with steel pipe concrete structures can be expected to improve the brittleness of prepacked aggregate concrete and solve the problem of volume shrinkage causing voids in steel pipe concrete components, realizing the development of prepacked aggregate concrete in structural engineering^27–29^. However, there is limited research in this area. As such, it is necessary to study the mechanical properties of PAC steel pipe concrete structures.

Therefore, it is necessary to further explore the influence of flowability and pouring methods on the compactness of PAC. It is also essential to conduct in-depth research on the mechanical properties of prefilled aggregate-filled steel pipe concrete, providing experimental data support for engineering applications. The gradation of pre-filled aggregates has a significant impact on the grouting behavior and mechanical properties of PAC. When the coarse aggregate porosity is excessively low, it becomes difficult to achieve dense grouting. Conversely, if the coarse aggregate porosity is excessively high, it increases the quantity of grouting material to fill the voids, ultimately increasing construction costs. Therefore, it is essential to maintain a suitable aggregate gradation that enables the successful grouting of a substantial portion of the grouting material. Currently, the author has completed an experimental study on the gradation of coarse aggregates using a ratio of 3:7 for coarse aggregates with particle sizes of 10 ~ 16 mm and 16 ~ 26.5 mm. The conditions result in an accumulation pore rate of 36.7%. Based on this, this article conducts experiments on matching the flowability of PAC with pouring methods and uses formulas, ultrasonic testing, and coring sampling to evaluate the compactness of different PAC specimens. For the well-performing PAC slurry mix and pouring methods, prefilled aggregate-filled steel pipe concrete is prepared, and its mechanical properties are studied.

## Materials and experimental program

### Materials

The materials used in this study included binders such as Ordinary Portland cement (OPC), fly ash, silica fume, fine and coarse aggregates and admixtures including expansive agents and superplasticizers. Class F fly ash with a loss on ignition of 4.98% is utilized. For silica fume, the silicon dioxide content is 93%, The chemical composition of the cementitious material is shown in Table 1. The maximum aggregate size of the manufactured sand is 2.36 mm. The particle size of crushed sandstone is between 10 and 25 mm, its porosity is 36.7% and the bulk density of coarse aggregate is 1.684. Moreover, the expansion agent is calcium oxide, and the superplasticizers with a 25% water reduction rate is used.

**Table 1:** Material chemical compositions (%).

Material	SiO_2_	Al_2_O_3_	Fe_2_O_3_	CaO	MgO	Na_2_O	K_2_O	SO_3_
Cement	20.31	4.80	4.99	65.5	1.30	0.15	0.40	2.10
Fly ash	58.00	30.00	4.30	1.50	2.80	3.20	–	/
Silica fume	95.72	/	0.48	1.62	0.21	/	/	

### Mixtures

**Figure 1:**
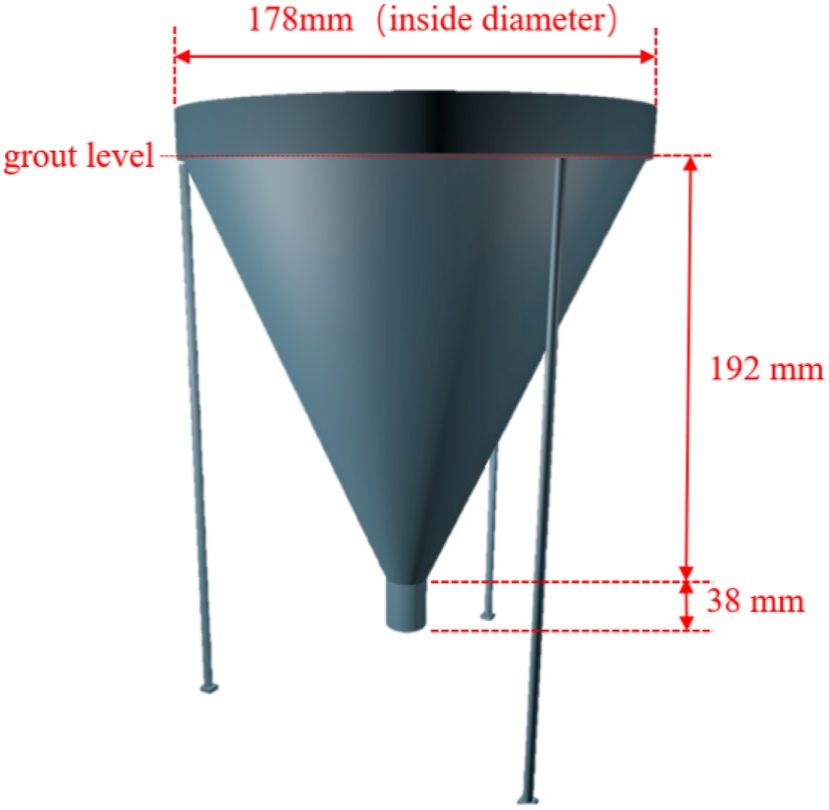
Dimensions of inverted cone.

Five different types of grouts with different fluidities are used to prepare PAC. Before forming the PAC specimens, all grouts are tested for flow time. The test procedure is performed according to GB/T 50448-2015^30^. The dimensions of the inverted cone are shown in Fig. 1. The PAC mixing ratios and grout fluidity are shown in Table 2.

**Table 2:** Mix design (kg/m^3^).

Mixture ID	Cement	Fly ash	SF^a^	EA^b^	Water	Sand	PSA^c^	Flow time/s
P_25_	1203	228	91	76	447	/	1.5%	25
P_35_	1264	240	96	80	420	/	1.3%	35
P_60_	1255	365	180	110	388	/	2.0%	60
M_30_	739	200	60	50	336	832	1.1%	30
M_40_	739	200	60	50	336	832	0.6%	40

^a^Silica Fumes, ^b^Expansion Agent, ^c^Superplasticizers.

### Sample molding method

**Figure 2:**
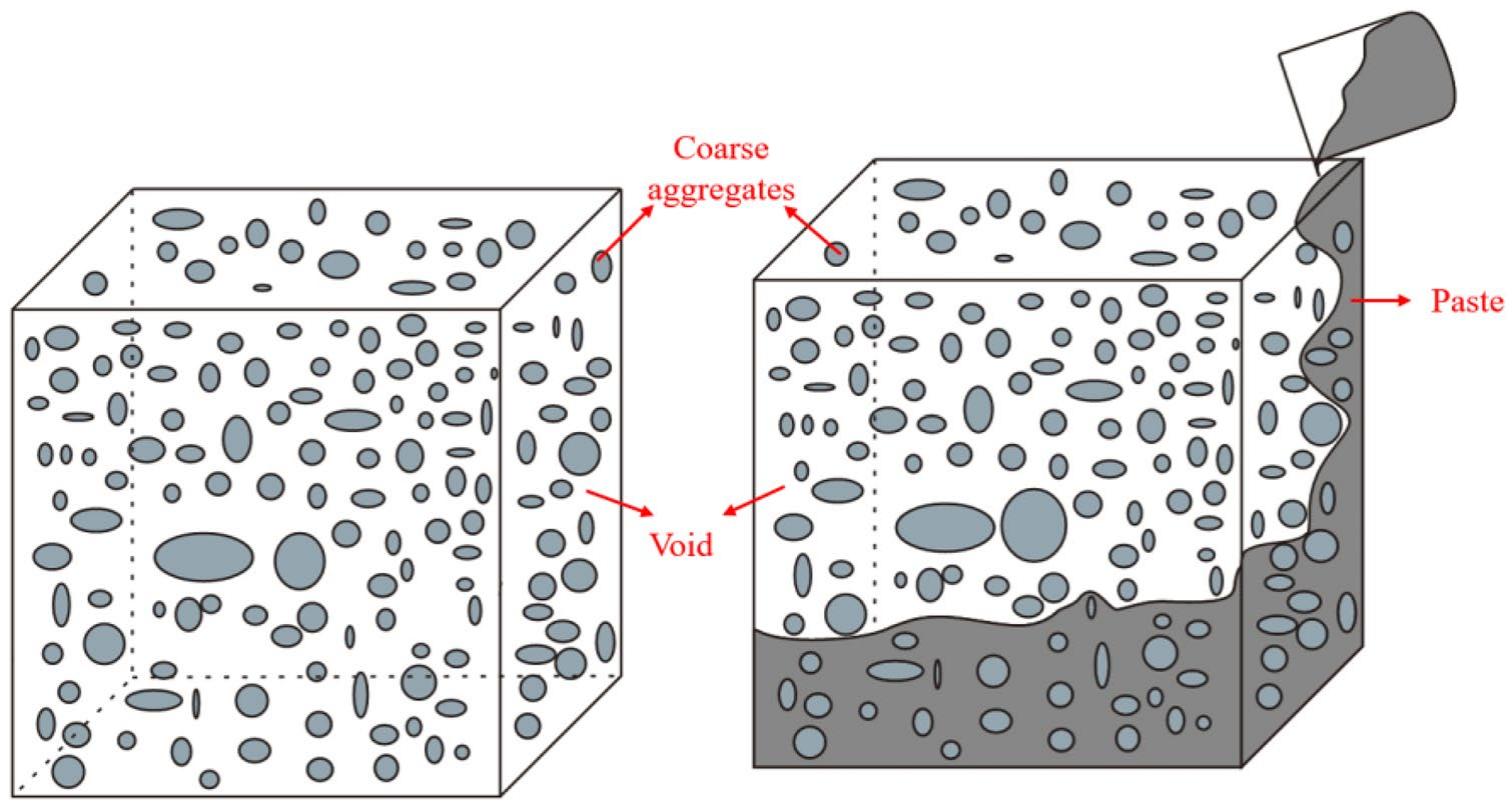
The schematic drawing of gravity pouring process.

**Figure 3:**
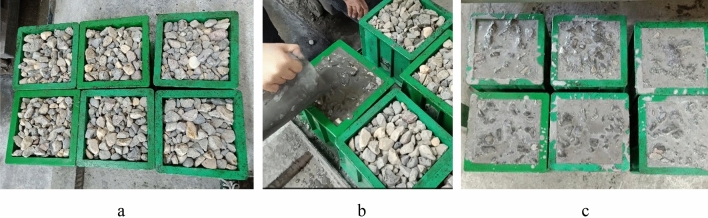
The actual gravity pouring. (**a**) Prefill coarse aggregate, (**b**) Gravity pouring, (**c**) Molding.

PAC preparation was carried out according to the following steps:

1. The grout was prepared according to the mixing ratios in Table 2. Measure the fluidity of the grout according to GB/T 50448-2015. After testing, the grout was reprepared and poured into 40 × 40 × 160 mm molds.
2. Coarse aggregates of 10–16 mm and 16–26.5 mm were put into 150 × 150 × 150 molds at a ratio of 3:7 by mass.
3. The grout was poured into 150 × 150 × 150 mm molds containing coarse aggregate by gravity pouring and pumping pouring. The gravity pouring and pumping pouring methods are as follows:

The gravity pouring method utilizes the gravity of the grout to penetrate the voids between the coarse aggregates. The gravity pouring method is shown in Fig. 2. As shown in Fig. 2, the grout is poured from one corner of the mold. The grout slowly penetrates the voids of the aggregate with gravity and then rises from the bottom to fill the entire mold. The actual gravity pouring is shown in Fig. 3.

**Figure 4:**
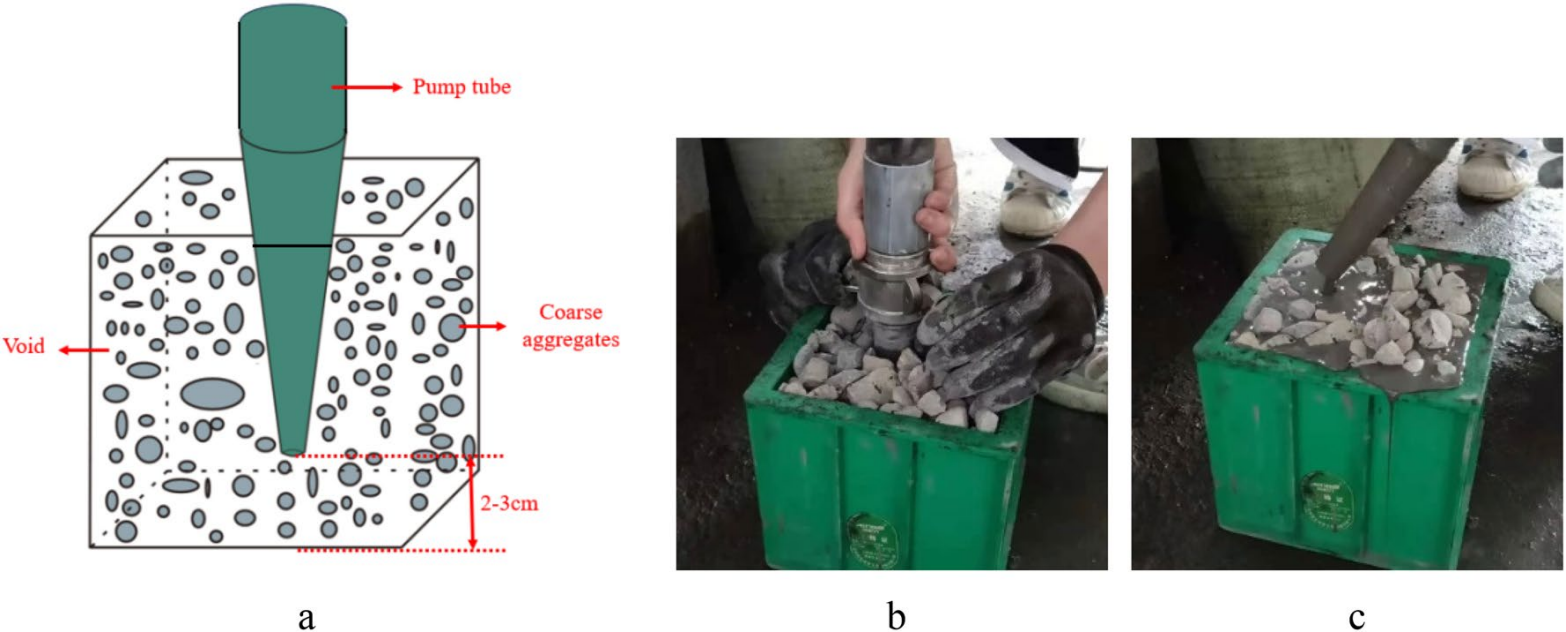
Pumping pouring process. (**a**) Pump pouring schematic, (**b**) Pump pouring, (**c**) Pouring completed.

The pump pouring method uses the pressure of the pump to inject the grout into the voids of the coarse aggregate. The grout is injected from the bottom upwards. The grout pipe is inserted approximately 2–3 cm above the bottom of the mold, as shown in Fig. 4. It should be noted for pump pumping that the pumping pressure may cause the aggregate to rise, and if necessary, a cover can be applied at the top of the template to prevent the aggregate from being flushed out. In addition, mortar is prone to segregation under pump pressure. To ensure the uniformity and consistency of the grout in the mold, the pump tip should be raised slowly while pumping.

### Experimental method

#### Mechanical properties

According to ASTM C349^31^, the compressive and flexural strengths of five different grouts were tested at 3 day, 7 day, and 28 day. The compressive strength of various PAC samples was evaluated and the strength value of each group is the average of three test specimens.

#### Compactness test

The compactness formula, ultrasonic pulse velocity and core sample integrity are three methods used for compactness evaluation.

1. It is a common way to calculate PAC compactness by formula. The formula is as follows:
  1 $$ D = \frac{V2}{{V1}} $$
  2 $$ V1 = \frac{{{\text{m}}1}}{\rho 1} $$
  3 $$ V2 = \frac{{{\text{m}}2}}{\rho 2} $$
  where D is compactness (%), V_1_ is the coarse aggregate void volume (m^3^), V_2_ is the actual grout pouring volume (m^3^), m_1_ is the mass of coarse aggregates, m_2_ is the mass of grout, ρ_1_ is the apparent density of the coarse aggregate, and ρ_2_ is the apparent density of the grout.
2. A ZBL-U5 nonmetallic ultrasonic detector is used for compactness evaluation of PAC. The 150 mm cube is divided into 5 sections along the horizontal and vertical axes, and the intersection of the horizontal and vertical measuring lines is the measurement point. The transducer is placed on both sides of the concrete sample, and the pulsed ultrasonic waves emitted by the transducer penetrate the concrete and are received by the receiving transducer on the opposite side of the specimen. Ultrasonic waves do not propagate in the air, so Vaseline is applied to the measurement point as a coupling agent to fill the gap between the transducer and the concrete surface to facilitate the transmission and transport of ultrasonic waves. The specific operation process is shown in Fig. 5. The measure points that are below 90% of the maximum velocity value at the measurement point may indicate internal defects, and these points are marked accordingly. Points within the range of 88–90% of the maximum velocity value are marked and combined with changes in other acoustic parameters to determine the presence of defects.

**Figure 5:**
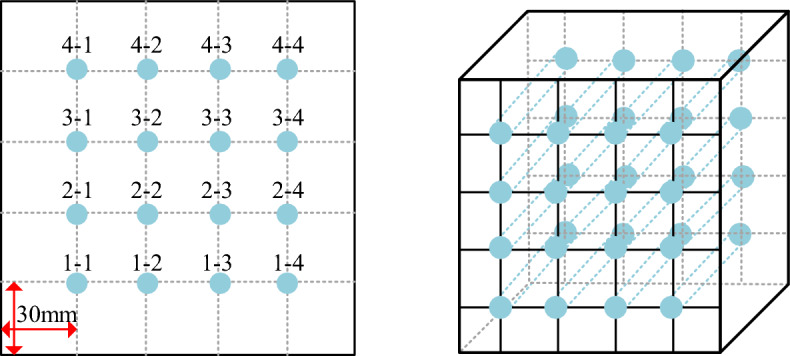
Ultrasonic detection points.
3. Core sample: according to JGJ/T384-2016^32^, the HZ-15 concrete core drilling machine was used to obtain core samples of the PAC specimens.

## Analysis and discussion of compactness

### PAC appearance fill integrity

#### Gravity pouring

**Figure 6:**
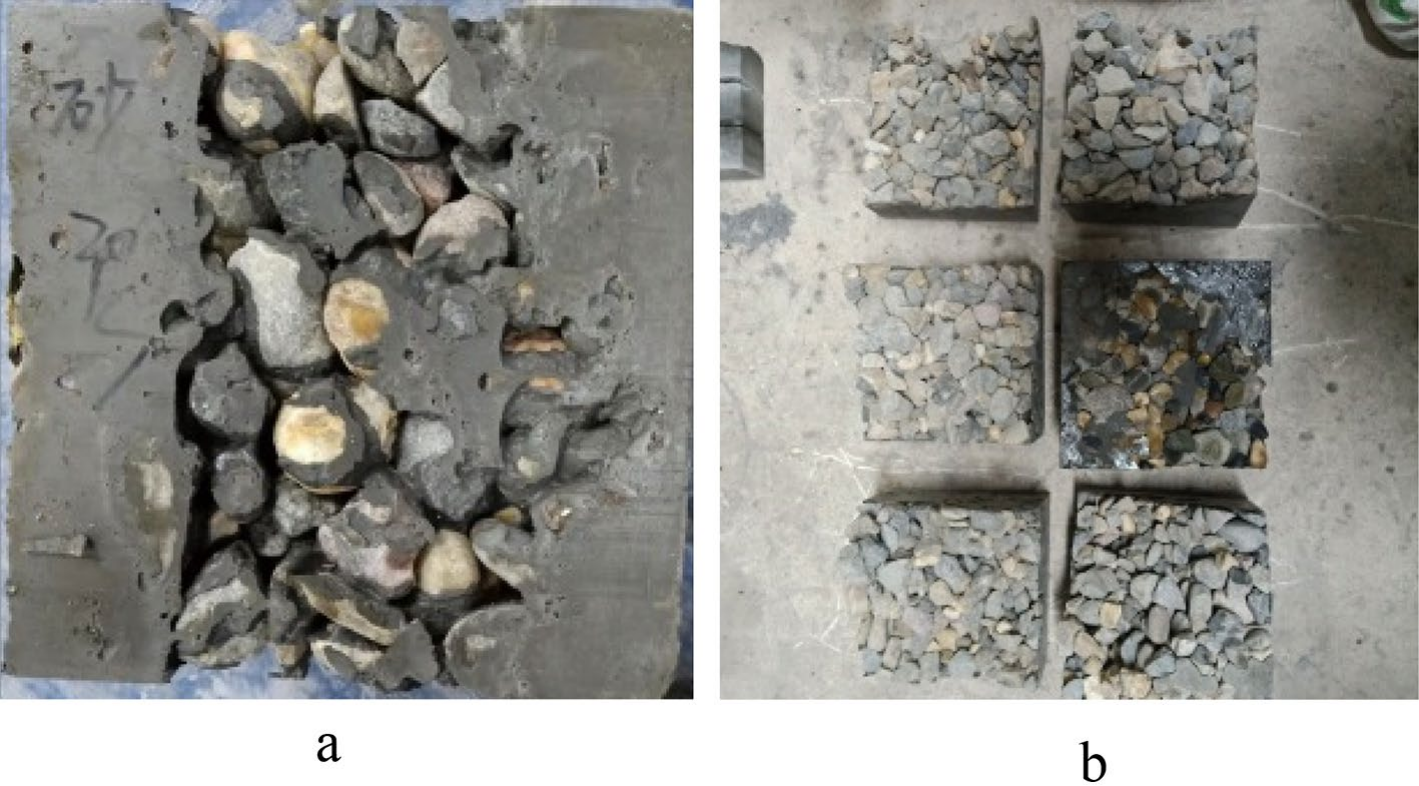
The failure PAC specimen with mortar gravity pouring. (**a**) Side view of the M30, (**b**) Bottom side of the M40.

Figure 6 shows the M30 and M40 gravity pouring forming specimens. A side view of M30 is illustrated in Fig. 6a. The maximum depth of M30 can reach the bottom of the formwork. However, there is severe leakage in the middle of the specimen, and obvious honeycomb defects exist. This is because to adjust the flowability of this group of mortar, too much water reducing agent was added. The separation of cement paste and fine aggregates leads to difficulty in grouting. The cement paste flows through coarse aggregates, reaching the bottom of the mold first during grouting. Then, the remaining grout is mixed with fine aggregate, which has difficulty passing through the gaps of coarse aggregates and accumulates in the upper half of the mold, resulting in weak areas in the middle of the specimen. Figure 6b is the bottom side view of M40. M32 and M40 did not penetrate the bottom of the formwork, and there was severe leakage of coarse aggregates on both the side and bottom of the specimens. The grout was poured onto the surface of the coarse aggregate, and the slurry slowly penetrated only by its own gravity. As the depth of the section increased, there might be situations where the grout could not completely penetrate the entire mold. The friction between the mortar and coarse aggregate is significant. The preparation of PAC with three groups of mortars by gravity pouring did not achieve a good filling effect. The greater flowability of the mortar can be further adjusted to achieve a better grouting effect. However, it also has higher segregation risks, which are also not conducive to PAC filling integrity.

**Figure 7:**
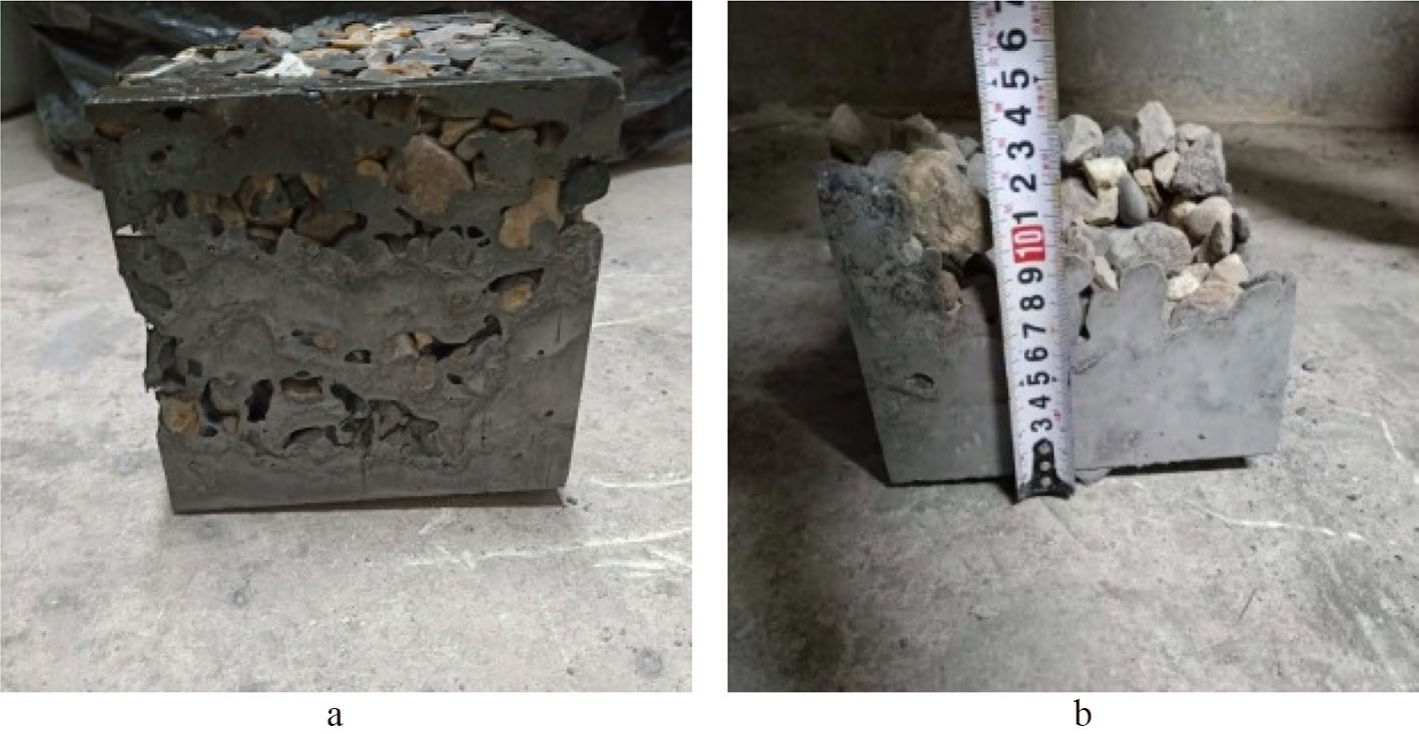
The pouring depth of the mortar test piece. (**a**) M30, (**b**) M40.

The comparison of the formed specimens of different flowability mortars is shown in Fig. 7. It can be observed that the higher the flowability of the mortar, the deeper the pouring depth of the specimens, and the better the filling degree of the mold. Among them, the pouring depth of M40 with the worst flowability was only approximately half of the mold height. M30 could penetrate the bottom of the mold, and the penetration ability of the grout was slightly improved.

**Figure 8:**
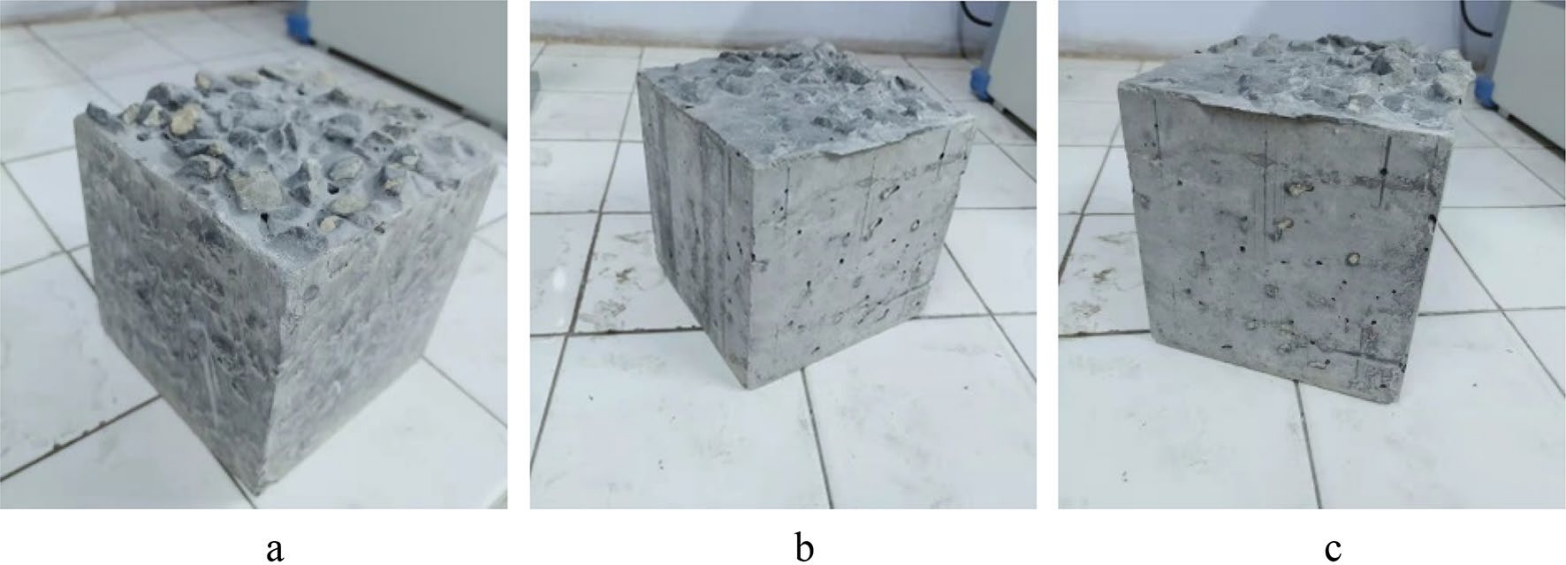
The PAC specimen with paste gravity pouring. (**a**) P25 specimen, (**b**) P35 specimen, (**c**) P60 specimen.

In contrast, paste is only composed of cementitious materials and water, and its flowability and stability are higher than those of mortar. Paste can quickly penetrate through the voids of the aggregate and uniformly cover the surface of the aggregate. The P25, P35, and P60 specimens formed by gravity pouring are shown in Fig. 8. All paste specimens are well filled without obvious defects. Combined with Figs. 6 and 8, it can be seen that the appearance integrity of P60 specimen is better compared with that of M30 specimen, and the flowability of P60 specimen is higher. The appearance integrity of the cement paste is superior to that of the mortar by gravity pouring.

**Figure 9:**
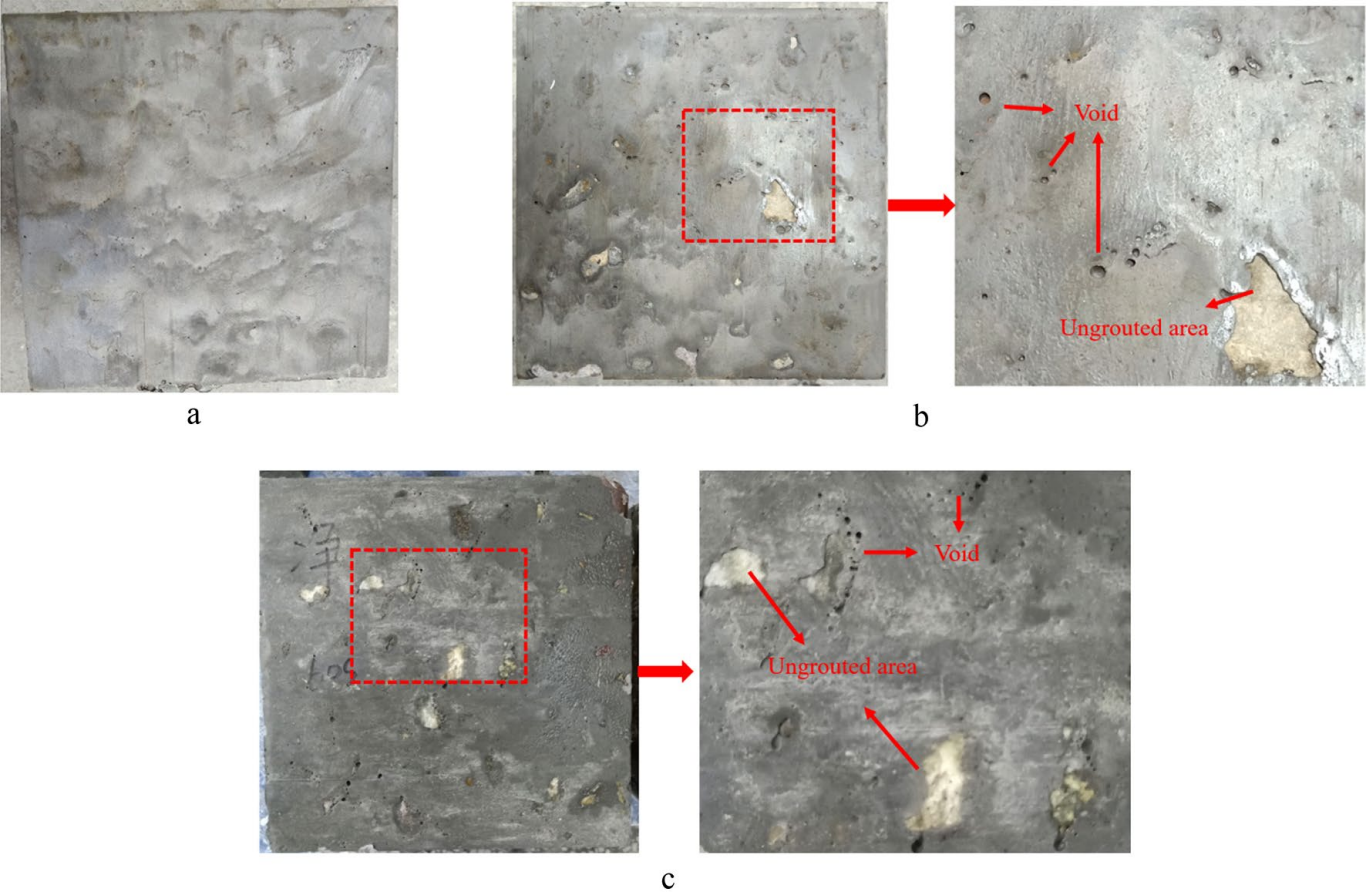
The side view of PAC specimen with paste gravity pouring. (**a**) P25 specimen, (**b**) P35 specimen, (**c**) P60 specimen.

The side view of the PAC specimen with paste gravity pouring is shown in Fig. 9. When the flowability of the paste is good, the side surface of P25 is smooth without holes. As the flowability decreases, there are more voids and ungrouted areas on the side surfaces of P35 and P60. Figure 9 shows concrete specimens formed through gravity casting, comprising P25, P35, and P60 grades. Visually, it is evident that the P25 specimen exhibits the least number of surface voids, while the P60 specimen displays the highest. In summary, for gravity pouring, improving the flowability of the grout can reduce the viscosity. It also reduced friction between grout and coarse aggregate and improved the filling effect.

#### Pump pouring

**Figure 10:**
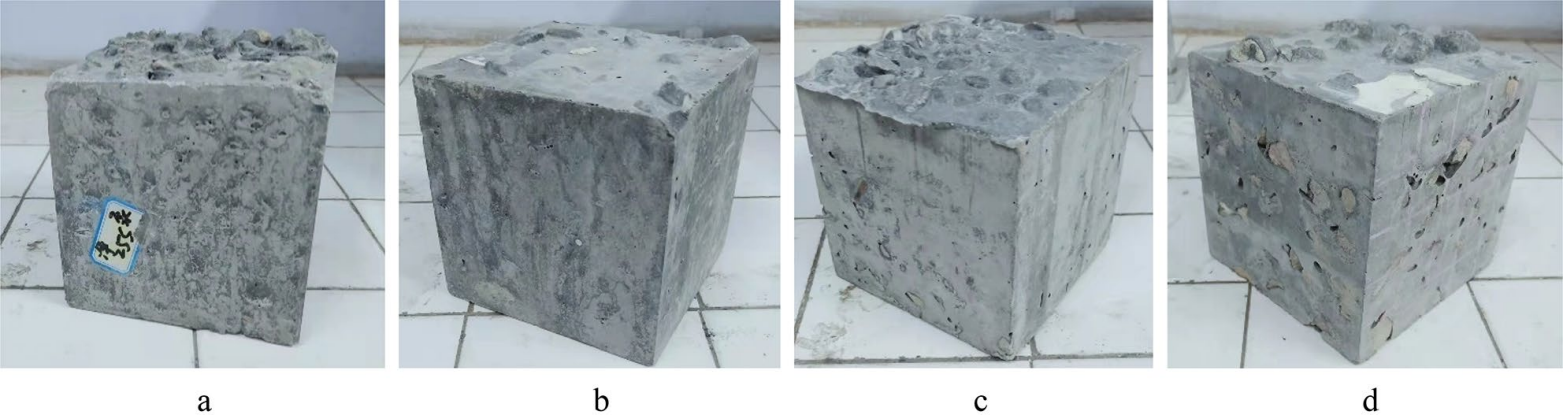
The PAC specimen with pump pouring. (**a**) P25 specimen, (**b**) P35 specimen, (**c**) M30 specimen, (**d**) M40 specimen.

Based on the results of gravity pouring, P25, P35, M30 and M40 were selected to prepare the PAC specimens with pump pouring. Note that P60 was not selected because despite the fact that the flow cone specimens of paste exceed those of mortar, the compactness of M40 specimens was consistently lower than those of P60 specimens formed through gravity placement regardless the gravity or pump method. This underscores the significant advantage of paste grouting materials in terms of enhancing the compactness of PAC. As shown in Fig. 10, all specimens are successfully formed without obvious defects. Pump pouring can greatly improve the limitations of gravity infusion. M40 successfully completed the pouring molding, but the surface had an ungrouted area. The M30 specimen with poor stability was also successfully poured and formed, with no voids in the middle of the specimen and no visual honeycomb defects. For the paste with good permeability, there is not much difference in appearance between the PAC specimens formed by gravity pouring and pump pouring.

### Compactness density

The calculated values of the compactness density are shown in Table 3. Regarding gravity pouring, the PAC specimens formed by paste have a higher compactness density than those formed by mortar. For the same grout, the better the fluidity is, the higher the compactness of the formed PAC specimen. The compactness of P25 is increased by 2.66% compared to that of P60. The compactness density of M30 increased by 38.15% compared to M60. For mortars with poor permeability, the increase in fluidity has a more obvious improvement effect on compactness density.

**Table 3:** Compactness density of the PAC formed by different grout/mold method.

Name	Paste wet density (kg/m^3^)	Mold method	Compactness (%)
P_25_	2000	Gravity	97.47
		Pump	98.98
P_35_	2128	Gravity	94.95
		Pump	98.25
P_60_	2174	Gravity	94.81
		Pump	/*
M_30_	2135	Gravity	76.37
		Pump	97.96
M_40_	2242	Gravity	38.22
		Pump	90.78

*/Means specimens were not molded.

The compactness of the PAC specimens formed by pump pouring is higher than that formed by gravity grouting. The compactness density of pump pouring P25, P35, M30 and M40 is increased by 1.51%, 3.3%, 22.04% and 52.56% compared to gravity pouring.

### Ultrasonic pulse velocity

The results of the ultrasonic pulse velocity test for PAC are shown in Tables 4 and 5. It can be observed that for the gravity grouting method, all three types of flowability mortar specimens indicate intact formation. Some individual measurement points of the P25 and P35 PAC specimens with better flowability may have defects, but their velocity values are still above 4000 m/s, indicating intact specimens. The ultrasonic waves of the concrete formed by gravity casting at P25 and P35 exhibit a higher level, indicating a superior compactness. In comparison to gravity casting, pump casting aids in enhancing the compactness of the specimens and elevating the ultrasonic wave velocity. The P60 specimens with slightly poorer flowability show complete formation, but their velocity values are lower than those of the other two groups of mortar specimens, indicating a decrease in compactness. The M30 specimens exhibit severe defects, with multiple points showing a significant decrease in velocity values. Most of these points have velocity values ranging from 1800 to 4000 m/s, which are lower than the speed at which ultrasonic waves propagate in concrete. For the pump pouring method, all PAC specimens show intact formation. The velocity values are higher compared to the PAC specimens formed using the gravity pouring method, indicating an improvement in compactness. Among them, the M30 specimens show the most significant improvement in compactness. The velocity of ultrasonic wave propagation is directly related to the compactness of the concrete. Higher velocity values indicate higher compactness, while lower velocity values indicate the presence of defects such as voids and honeycomb surfaces, which compromise the overall integrity of the concrete. In these cases, the sound waves cannot propagate in a straight line through the voids. They can only be transmitted by bypassing the defect, resulting in an extended propagation path, longer transit time, decreased velocity values, decreased waveform amplitudes, and waveform distortion or superposition. Ultrasonic pulse velocity testing provides a more intuitive and convincing evaluation of the compactness of PAC and can serve as a primary testing method in the future.

**Table 4:** Pulse velocity of the PAC measuring point 1 and 2 (km/s).

Name	Mold method	Detection position							
		1–1	1–2	1–3	1–4	2–1	2–2	2–3	2–4
P_25_	Gravity	4.767	4.669	4.871	4.630	4.688	4.688	4.611	4.808
	Pump	4.978	4.871	4.852	4.861	4.850	4.978	4.850	4.789
P_35_	Gravity	4.855	4.871	4.891	4.830	4.833	4.652	4.732	4.871
	Pump	4.927	4.874	4.811	4.808	4.916	4.850	4.935	4.935
P_60_	Gravity	4.728	4.650	4.768	4.630	4.669	4.656	4.767	4.732
	Pump	/	/	/	/	/	/	/	/
M_30_	Gravity	4.213	4.261	3.866	4.31	4.076	3.571	3.261	2.072
	Pump	4.831	4.835	4.729	4.710	4.650	4.732	4.728	4.749
M_40_	Gravity	/	/	/	/	/	/	/	/
	Pump	4.786	4.623	4.845	4.521	4.284	4.415	4.215	4.654

**Table 5:** Pulse velocity of the PAC measuring point 3 and 4 (km/s).

Name	Mold method	Detection position							
		3–1	3–2	3–3	3–4	4–1	4–2	4–3	4–4
P_25_	Gravity	4.688	4.537	4.611	4.632	4.611	4.632	4.669	4.913
	Pump	4.727	4.850	4.852	4.913	5.001	4.874	4.809	4.790
P_35_	Gravity	4.872	4.654	4.679	4.701	4.748	4.750	4.689	4.749
	Pump	4.788	4.934	4.852	4.789	4.893	4.773	4.728	4.956
P_60_	Gravity	4.693	4.809	4.650	4.651	4.728	4.768	4.669	4.713
	Pump	/	/	/		/	/	/	/
M_30_	Gravity	2.451	2.451	2.586	3.505	3.319	3.571	1.829	3.205
	Pump	4.728	4.654	4.615	4.520	4.659	4.708	4.631	4.669
M_40_	Gravity	/	/	/		/	/	/	/
	Pump	4.456	4.823	4.810	4.554	4.845	4.514	4.365	4.154

The PAC specimens with P_60_-pump pouring were molded. The PAC specimens with M_40_ gravity pouring were irregularly shaped.

### Core sample integrity

**Figure 11:**
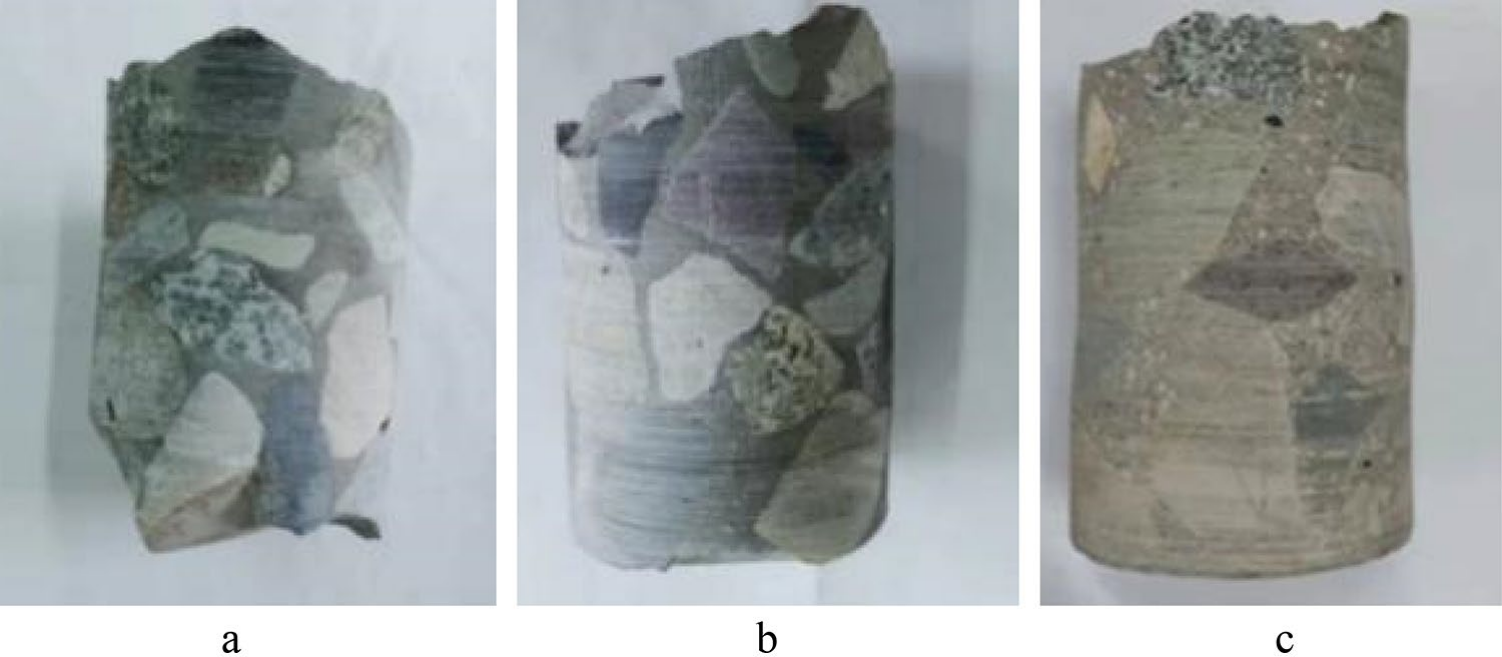
Pump pouring core sample. (**a**) P25, (**b**) P35, (**c**) M30.

Figure 11 shows the core sample of the PAC specimen formed by pump grouting, which appears intact without evident holes or cracks, indicating good compactness consistent with the compactness calculation values and ultrasonic pulse testing results. Figure 11c also reveals fine aggregates suspended in the slurry, with mutual interlocking between them and between the fine aggregates and coarse aggregates, indicating that the fine aggregates further enhance the constraint effect of the slurry on the coarse aggregates, strengthening the interface bonding between them and inhibiting the shrinkage of mortar and PAC.

**Figure 12:**
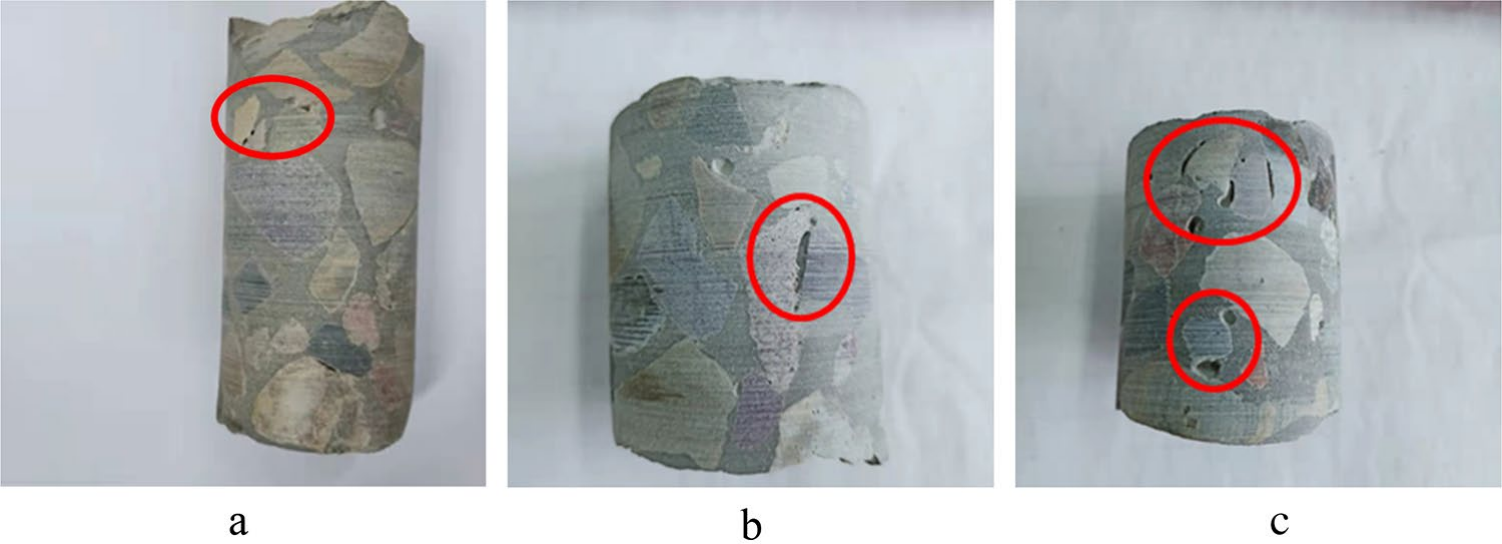
Gravity pouring core sample. (**a**) P25, (**b**) P35, (**c**) P60.

The core sample of the PAC specimen formed by gravity pouring is shown in Fig. 12. The compactness of the PAC specimen formed by a poorer flowability mixture is lower, and there are more voids and regions without grout. The sampling of core samples provides a visual assessment of the compactness of concrete. However, for large-volume engineering projects, the representativeness of core samples is limited, and the core should be filled and reinforced after coring, so the detection method of drilling core sampling still has some shortcomings.

## Analysis and discussion of mechanical properties

### Mechanical properties of the grout

**Figure 13:**
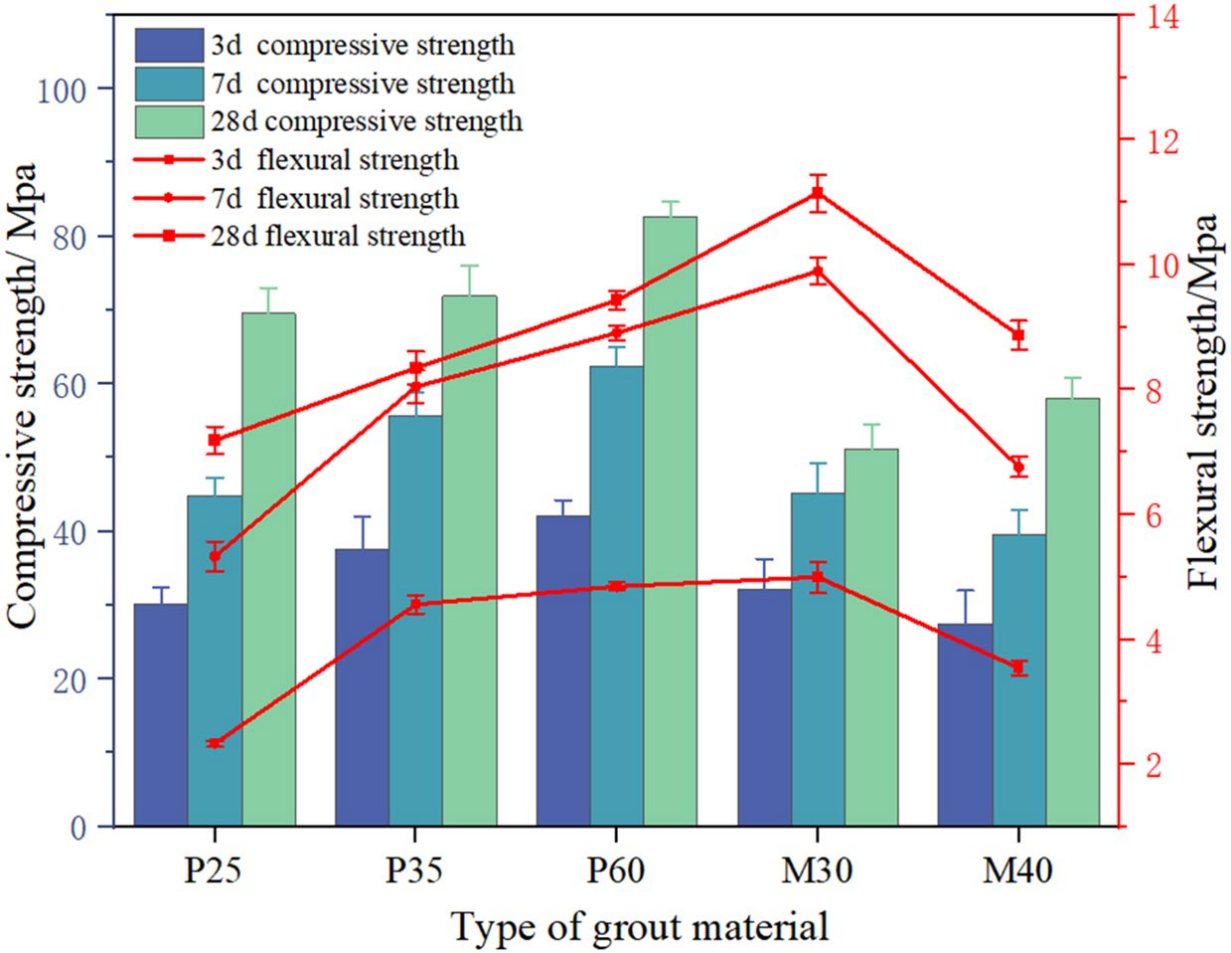
Mechanical properties of grout.

The mechanical properties of the grout are shown in Fig. 13. The flexural and compressive strengths of the paste (P25, P35, P60) increase as the water-cement ratio decreases. From the compressive strength results of the mortar at 28 days (M30, M32, M40), it is evident that the addition of a water reducer can enhance the compressive strength when other parameters of the mix design are held constant. However, when the water reducer is overdosed, it no longer contributes to the strength.

### Mechanical properties of the PAC

**Figure 14:**
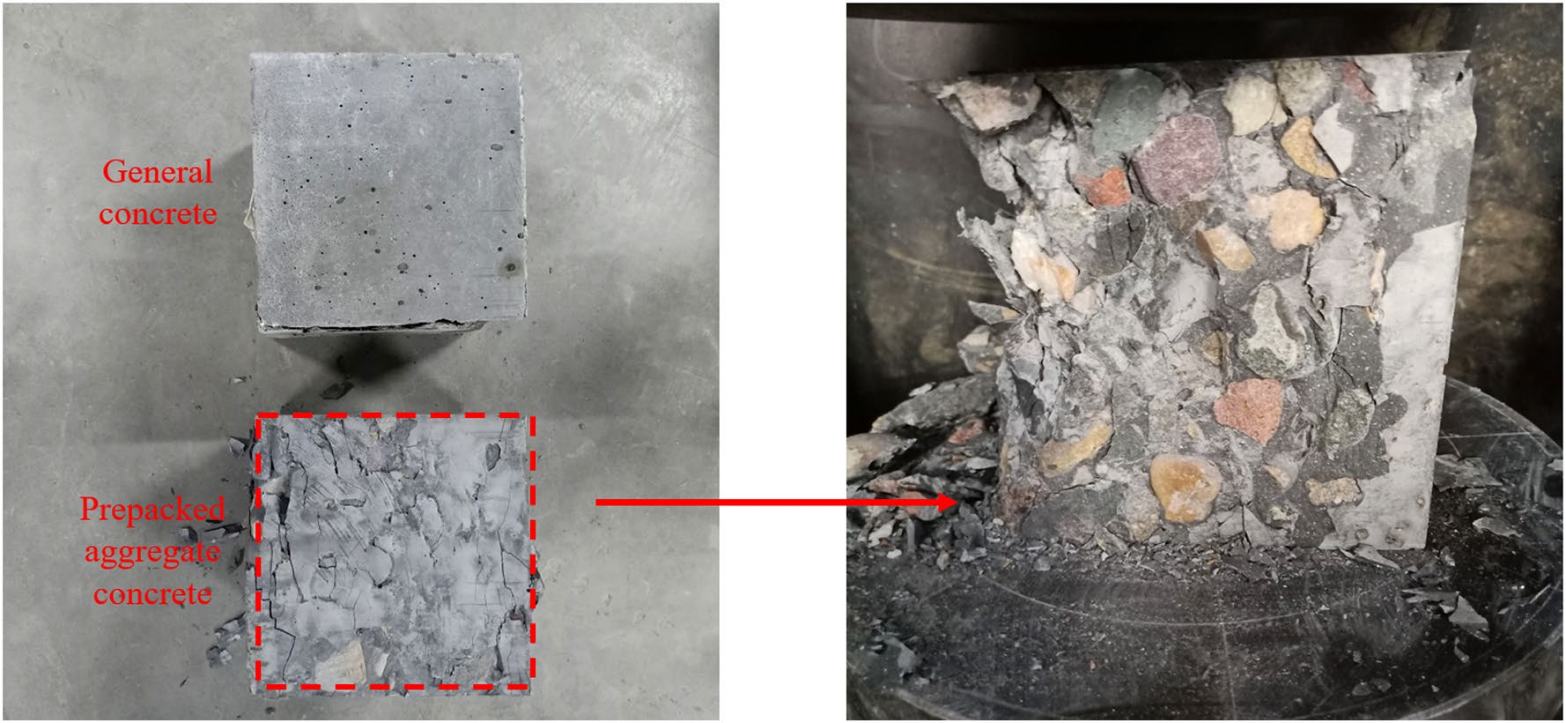
Compressive failure mode.

The compressive failure morphology of the PAC specimens is illustrated in Fig. 14. The axial compression failure of the PAC is accompanied by the sound of aggregate crushing, and the surface morphology of the failure is different from that of conventional concrete. The surface of PAC is covered with cracks formed by the tearing of the mortar from the aggregate. The failure of PAC is caused by the failure of the bond between the mortar and aggregate surfaces, which is induced by the crushing of coarse aggregate particles. This observation from the side view confirms the skeletal role of PAC coarse aggregate.

**Figure 15:**
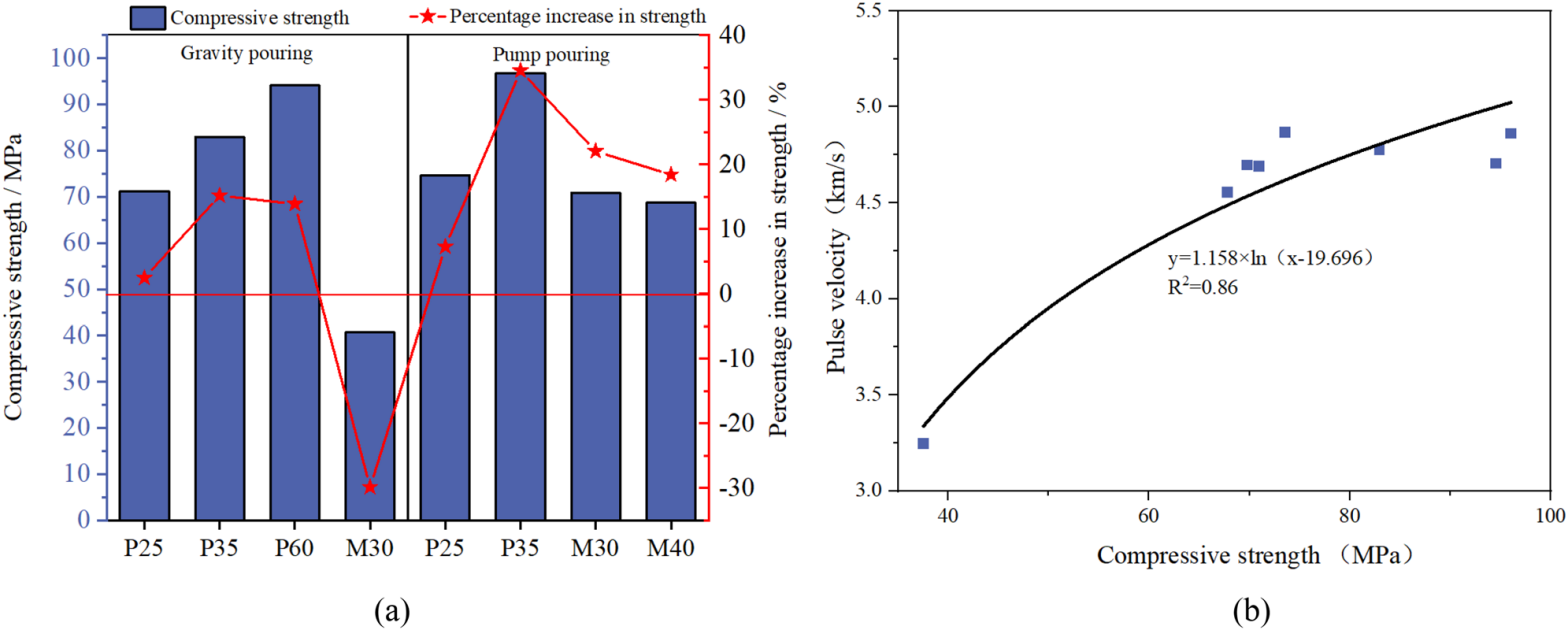
(**a**) 28 day compressive strength of PAC, (**b**) relationship between PAC compressive strength and pulse speed.

Figure 15a shows the 28 day compressive strength and percentage increase in strength (compared to the 28 day compressive strength of grout) for different PACs. The pouring method has little influence on the compactness and mechanical properties of PAC specimens with good flowability grout. The 28 day compressive strength PAC of P25-gravity pouring and P25-pump pouring show growth of 2.46% and 7.28%, respectively, compared to grout specimens. For PAC specimens formed by M30, the strength difference between the PAC specimens manufactured by the two pouring methods reaches 30.1 MPa, indicating that compactness directly affects the mechanical properties of PAC. Except for the PAC specimens manufactured by M30-gravity pouring, the compressive strength of the PAC specimens with good compactness shows an increase compared to the compressive strength of the grout. The highest increase reaches 34.5%. Moreover, Table 3 indicates a decreasing trend in compactness for P25, P35, and P60, which is in line with the observable characteristics depicted in their respective appearances. However, the reason behind the non-decreasing trend in compressive strength for P25, P35, and P60, despite their decreasing compactness in Fig. 15a, lies in the fact that the flow time of these grades is regulated by the composition of admixtures and binding materials. Adjusting the admixture dosage to alter the flow time of grouting materials can lead to segregation and other issues. Although P60 exhibits a slower flow time and a relatively lower compactness upon setting, it contains the highest amount of cementitious materials, resulting in an increased production of hydration products that provide higher strength to the concrete. This observation aligns with the findings presented in Fig. 13.

Therefore, only PAC with good compactness can ensure the bonding between grout and aggregate surfaces and effectively develop strength. The relationship between the compressive strength and pulse velocity of PAC is illustrated in Fig. 15b, showing a correlation that conforms to a logarithmic function. When the strength of the specimens decreases to 37.5 MPa, the ultrasonic pulse velocity experiences a significant reduction of 31.5%. Therefore, ultrasonic testing is an effective method for detecting substantial defects in concrete as indicated by the notable decrease in pulse velocity when the compressive strength reaches this threshold.

## Mechanical properties of the PASPC

This paper further investigates the mechanical properties of prepacked aggregate steel pipe concrete (PASPC). According to the results of 3.1, 4.1 and 4.2, the mix ratios P_25_ and M_30_ were formed to carry out the load‒displacement, strain and transverse deformation coefficient tests of steel pipe concrete. The PASTC with mix ratio P_25_ was formed by gravity pouring and pump pouring. The PASTC with mix ratio M_30_ was formed by pump pouring only. Conventional steel pipe concrete (CSPC) is formed using conventional techniques. The specific molding parameters are shown in Table 6.

**Table 6:** Basic parameters of steel pipe concrete.

Specimen number	Pipe size*/mm	Pipe type	Molding method	Grout mix ratio	Grout type
PASTC-GP_25_	317.5 × 127 × 4.5	Q235	Gravity pouring	P_25_	Cement paste
PASTC-PP_25_	317.5 × 127 × 4.5	Q235	Pump pouring	P_25_	Mortar
PASTC-PM_30_	317.5 × 127 × 4.5	Q235	Pump pouring	M_30_	Mortar
CSPC	317.5 × 127 × 4.5	Q235	/	/	/

*Pipe sizes are height × outside diameter × wall thickness.

### Results of the load‒displacement, strain and transverse deformation coefficients

**Figure 16:**
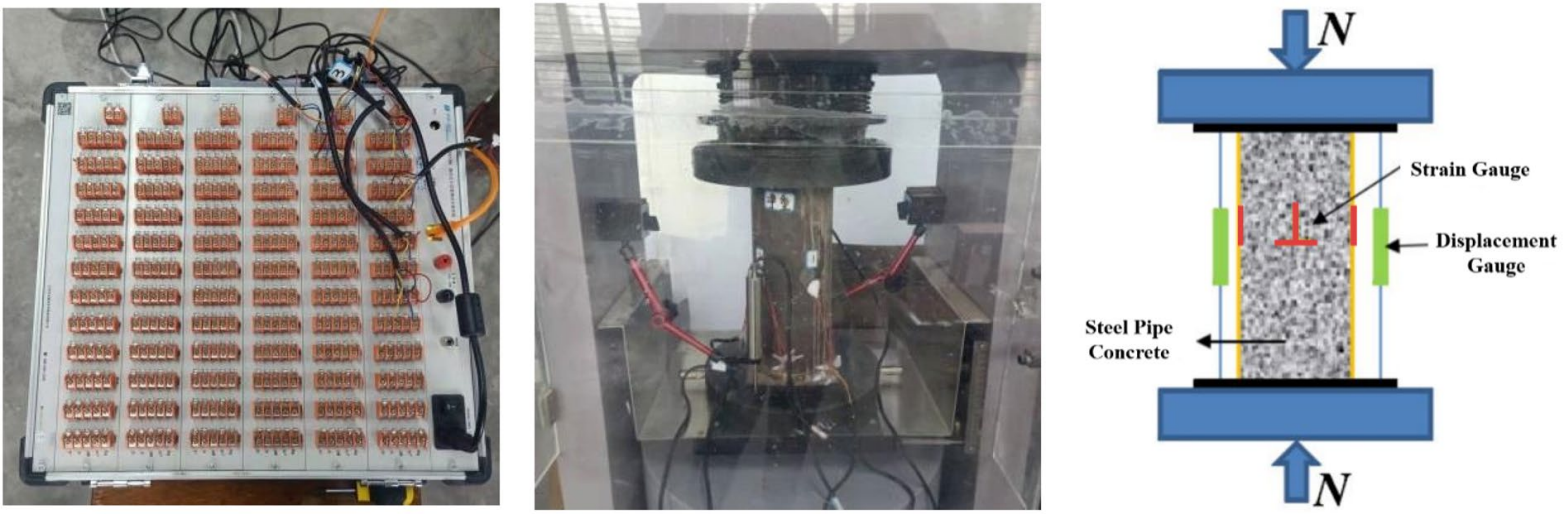
Loading force test procedure.

Figure 16 shows the process of the steel pipe concrete compression experiment. The vertical loads, displacements, and strains of the members were measured during the test. The outer surface of the pipe is divided into four areas at 90° intervals. Eight strain gauges were arranged in each area. The transverse strain was tested by 4 horizontally arranged strain gauges. The vertical strain was tested by 4 vertically arranged strain gauges. Two displacement gauges are arranged on the side of the pipe to measure the change in axial displacement.

**Figure 17:**
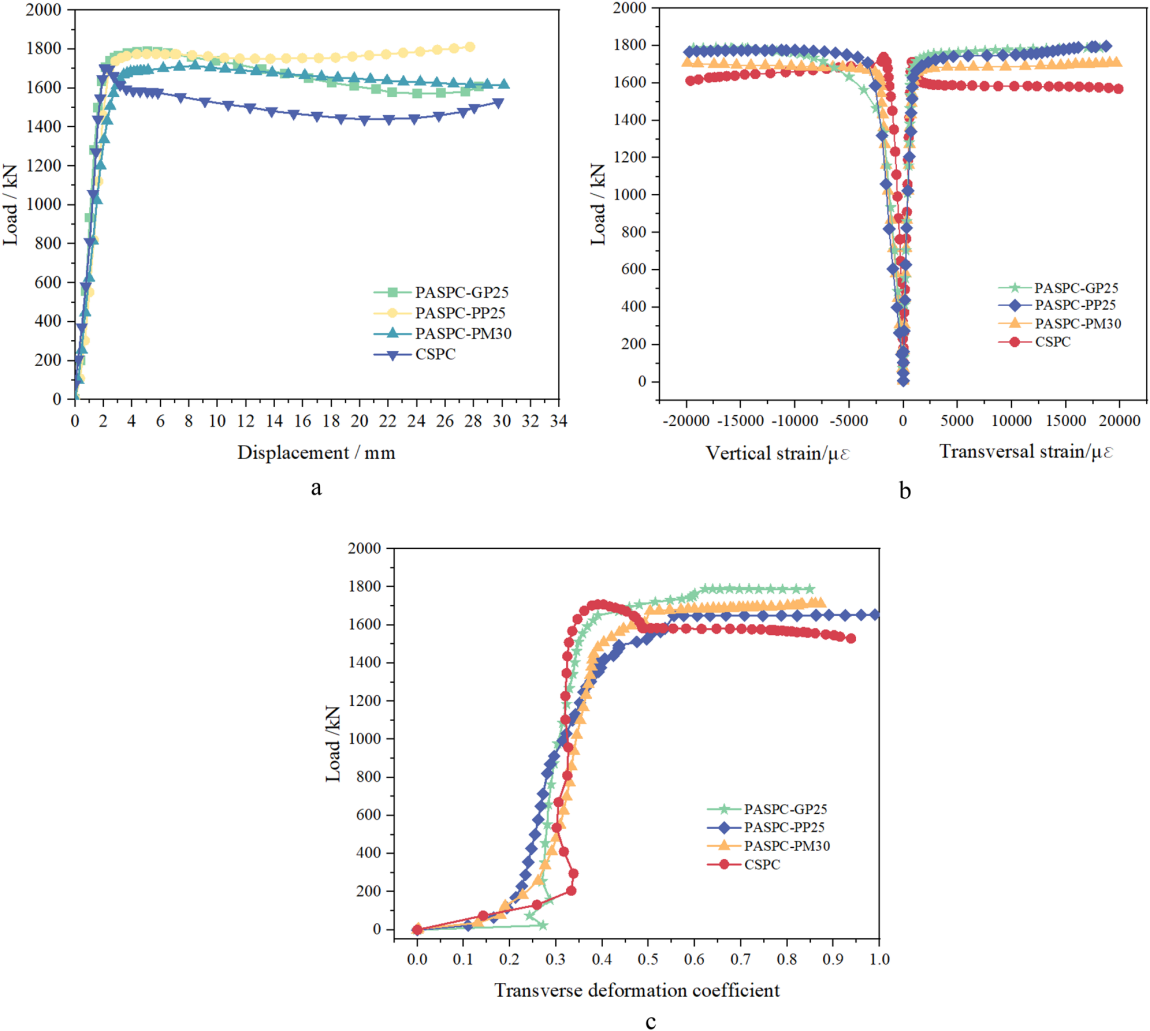
Load‒displacement, strain and transverse deformation coefficient curves. (**a**) load‒displacement, (**b**) load–strain, (**c**) load–transverse deformation coefficient.

Figure 17a shows the load‒displacement relationship. All the PASPC specimens have similar load‒displacement curve trends. The loading process can be divided into three stages: elastic stage, elastic‒plastic stage and plastic-enhanced stage. In the elastic phase, the compressive load and displacement of the specimen increase approximately linearly. In the elastic‒plastic phase, the load‒displacement curve is nonlinear. The slope of the curve decreases gradually; when the slope approaches 0, the specimen reaches the ultimate bearing capacity. During the plastic-enhanced stage, the axial compressive load capacity of PASPC basically does not decrease and shows good ductility. This is significantly different from the CSPC. The CSPC specimens show a decreasing and then increasing curve during the plastic-enhanced stage.

Figure 17a and b shows the load-strain and transverse deformation coefficient plots. where the transverse deformation coefficient is calculated as follows:

4 $$ \alpha {\text{trc = }}\left| {\frac{{\varepsilon {\text{t}}}}{{\varepsilon {\text{v}}}}} \right| $$

where $\varepsilon {\text{t}}$ is the vertical strain ($\mu \varepsilon$) and $\varepsilon {\text{v}}$ is the transverse strain ($\mu \varepsilon$).

The load-strain curves of the PASPC specimens basically show an increasing, stabilizing, and increasing again trend. At the end of the elastic stage, the specimens were stressed at approximately 80% of the peak load, and the strains ranged from 1300 to 1500. The transverse deformation coefficient fluctuates within 0.2 ~ 0.3. At this time, the Poisson's ratio of concrete is smaller than the Poisson's ratio of steel pipe, and the two have a tendency to separate. In the elastic‒plastic stage, the core concrete is under pressure, and microcracks continue to develop. When its transverse deformation coefficient exceeds the Poisson's ratio of steel, the concrete deformation is constrained by the steel pipe. As shown in Fig. 17c, the transverse deformation coefficient begins to grow. At this time, the core concrete is subjected to a three-way force, and the steel pipe is subjected to a two-way force. In the plastic-enhanced stage, the steel pipe cannot effectively restrain the transverse deformation of concrete. The load remains stable, but the strain grows very fast, and the transverse deformation coefficient grows linearly. Bulging and buckling of the steel pipe occurs. Overall, all steel pipe concrete specimens with sufficiently large hoop coefficients showed good ductility.

### Axial compressive capacity of PASPC

Table 7 demonstrates the results of the nominal load carrying capacity ($N{\text{c}}$) and the magnitude of capacity enhancement (SI) and hoop index ($\xi$) for steel pipe concrete. $N{\text{c}}$ and $SI$ and $\xi$ are calculated as follows:

5 $$ N{\text{c}} = A{\text{sfy}} + A{\text{cfc}} $$

6 $$ SI = \frac{N\max }{{N{\text{c}}}} $$

7 $$ \xi = \frac{{A{\text{sfy}}}}{{A{\text{cfc}}}} $$

where Ac is the cross-sectional area of the concrete (mm^2^), Ac is the cross-sectional area of the steel pipe (mm^2^), and f_y_ is the yield strength of steel (406.5 MPa), and fc is the concrete axial compressive strength (MPa).

**Table 7:** Axial compressive load capacity.

Specimen number	fc/MPa	N_max_*/kN	$\xi$	Nc/kN	SI
PASPC-GP_25_	41.6	1745.2075	1.55	1157.323	1.510
PASPC-PP_25_	53.4	1774.1435	1.21	1287.301	1.375
PASPC-GM_30_	49.8	1694.4700	1.29	1247.952	1.355
CSPC	48.7	1727.8075	1.32	1235.929	1.400

*N_max_ is the maximum value of the actual axial compressive capacity.

The compressive strength of steel pipe concrete is significantly increased under the constraints of the steel pipe. The axial compressive capacity of steel pipe concrete is no longer a simple iteration of the two. PACFST-GP_25_ members have the largest SI. The larger the hoop index is, the better the concrete restraining performance of the steel pipe, and the greater the increase in axial compressive capacity. PACFST-PP_25_ has the smallest hoop index. However, its core concrete strength and SI are similar to those of the other groups. The compactness of the specimens formed by paste is higher than that of the specimens formed by mortar with the same pouring method. The SI index of PASPC is closely related to the compactness of concrete in the pipe and the type of grout.

### Autogenous shrinkage of PASPC

**Figure 18:**
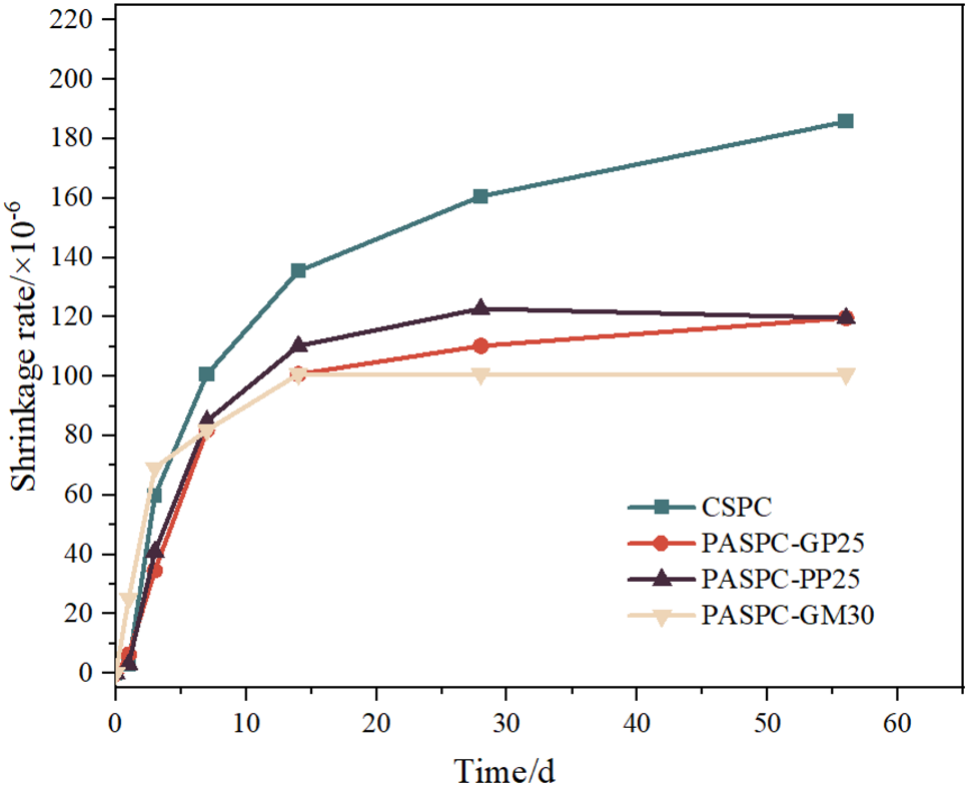
Autogenous shrinkage of steel tube concrete.

All steel tube concrete components were tested for their autogenous shrinkage under sealed conditions, and the test results are shown in Fig. 18. The shrinkage of prefilled aggregate steel tube concrete was significantly lower than that of conventional steel tube concrete (CSPC), with a maximum reduction of 54.23%. Prefilled aggregate steel tube concrete has a high volume fraction of coarse aggregates in the pipe, resulting in less cement usage. The point-to-point contact of the aggregates further limits the shrinkage of the grout. In addition, the shrinkage of steel tube concrete components with net grout placement is higher than that of steel tube concrete components with mortar placement. This is because the fine aggregates can have a limiting effect in the cement slurry. Overall, the volume stability of prefilled aggregate steel tube concrete is improved compared to conventional steel tube concrete, effectively reducing autogenous shrinkage effects.

## Conclusions

To explore the correlation between PAC compactness and pouring methods, this study used different types and different fluidity values of grout to form PAC specimens. The coupled effect of pouring methods and grout performance on the compactness of PAC was considered. Moreover, the application feasibility of PAC based steel tube concrete was investigated. The main observations are as follows:

1. The size of aggregate particles and the fluidity of the grout directly affect the choice of grout pouring method. The gravity pouring method is suitable for grout with good flowability. Paste is more suitable for gravity pouring than mortar, as even paste with a flow cone time of 60 s can still be formed completely. The compactness of the PAC specimens using the pumping pouring method is higher than that of specimens prepared with the gravity method. With the pumping pouring method, the prepacked aggregate concrete with a 28-day compressive strength of 96.1 MPa using the cement paste by pumping was successfully prepared.
2. Evaluating the compactness of PAC specimens can be achieved through various methods, including the observation of the completeness of specimen, calculations of density, ultrasonic pulse testing, and core sampling. Among these techniques, ultrasonic pulse testing stands out as a non-destructive approach, while core sampling represents a semi-destructive method. Notably, ultrasonic pulse testing offers unparalleled convenience and high accuracy compared to core sampling, leading to the preferred choice for assessing the compactness of PAC specimens.
3. All steel tube concrete exhibited a typical waist drum failure mode. The axial load–displacement curve of the short columns made of PASPC can be roughly divided into the elastic stage, elastic–plastic stage, and plastic strengthening stage. The elastic stage of the load–displacement curve for PASPC is longer. The curve shows almost no decline in load-strain, demonstrating excellent ductility. Substituting PAC for conventional concrete in the preparation of steel tube concrete columns presents significant economic and environmental benefits.

## Data Availability

The datasets used and/or analysed during the current study available from the corresponding author on reasonable request.
